# Stroke and Telerehabilitation: A Brief Communication

**DOI:** 10.2196/18919

**Published:** 2020-07-17

**Authors:** Ayisha Bashir

**Affiliations:** 1 Department of Biomechanics University of Nebraska Omaha, NE United States

**Keywords:** telerehabilitation, rehabilitation, nursing, stroke, telehealth

## Abstract

This rapid communication highlights stroke telerehabilitation, a health care service that provides daily monitoring of the care of patients recovering from stroke, delivering convenient and immediate feedback for patients, family, and caregivers. The delivery, management, and coordination of nursing care services, provided via telecommunications technology, is a convenient method of delivering health care to patients recovering from stroke. It is important to assess the service quality of the telehealth process and to establish the role of telehealth nursing and related technologies in the care of patients recovering from stroke. Studies show that even though both health professionals and participants have reported high levels of satisfaction and acceptance of telerehabilitation interventions, the quality of the evidence on telerehabilitation in poststroke care remains low. Conducting a quality study of telehealth rehabilitation for patients recovering from stroke will help assess if home health agencies with telehealth capabilities caring for patients recovering from stroke and patients with chronic diseases can provide quality care to patients in their home and fill this health care gap. Patients that are severely handicapped and impaired and unable to reside in their home environment are not included in telerehabilitation services provided by the home care agency. It would be informative to study the benefits of telerehabilitation and the care provided to patients recovering from stroke within nursing homes, given the need for social distancing to reduce disease transmission during the current coronavirus disease (COVID-19) global health pandemic. Using telerehabilitation would mean that patients have a lower risk of exposure to infectious agents. Further research into telehealth interventions and stroke management in home care is crucial.

## Background

**Figure 1:**
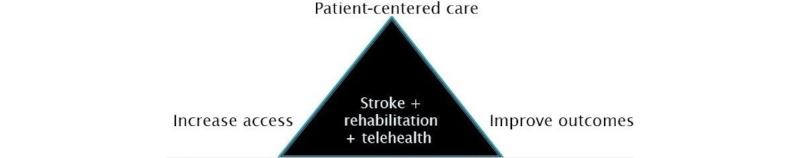
Goals for telerehabilitation for poststroke care.

Telerehabilitation is an emerging method of health care delivery through which medical rehabilitation care can be provided from a distance, using telehealth to provide remote health care [1,2]. In stroke care, immediate assessment and treatment are essential to reduce the risk of death and disability [3]. However, many patients do not receive adequate treatment due to a lack of specialist services. Technological development, telehealth techniques, and telehealth stroke rehabilitation may facilitate future telehealth projects, improve care team satisfaction, and allow patient management to continue at home [2,3]. The use of telehealth and telecommunications technologies promotes self-care and well-being and enhances positive health outcomes in people with stroke, peripheral artery disease, and severe disabilities and is favored by their family caregivers [3,4]. In addition, telerehabilitation aims to provide more patient-centered care and greater access (Figure 1). The importance of matching technology to the needs of this population and the lessons learned from these investigations should be addressed. This viewpoint highlights findings from a pilot study related to telehealth nursing effectiveness for patients with chronic diseases [4] and applies these notions toward future investigations related to stroke telerehabilitation. Numerous studies have assessed the cost-effectiveness and patient acceptance of telehealth methods of service delivery, yet the perspectives of service providers and caregivers delivering stroke care have not been studied [5,6].

## Discussion

### Overview

Research findings indicate that even though health professionals and participants have reported high levels of satisfaction and acceptance of telerehabilitation interventions, the overall quality of the evidence on telerehabilitation in poststroke care remains low [2,7].

In the article “Perspectives of Nurses Toward Telehealth Efficacy and Quality of Health Care: Pilot Study,” the authors examined whether telehealth technology impacts the perceived level of internal service quality delivered by the nurses of the relevant telehealth organization, the Visiting Nurse Association (VNA). A similar study of telehealth rehabilitation for patients recovering from stroke aimed to assess if home health agencies with telehealth capabilities caring for patients with chronic diseases and those receiving stroke rehabilitation can take care of patients in their home setting, thereby filling this gap [3]. Telerehabilitation for patients recovering from stroke is perceived as providing convenience and a sense of security to the patient, caregivers, and family members, allowing timely nursing interventions under supervised physician care [2,7]. Caring for the needs of the patients recovering from stroke represents a significant financial and emotional burden. Telehealth interventions can improve self-management, communication, and the engagement of caregivers involved in the long-term care of these patients [3].

Patients with chronic disease and patients recovering from stroke are generally provided with services including companion care, infusion pharmacy, home care, home health technology, hospice, and palliative care. Organizations using telehealth nursing services monitor the patient through assessment and the collection of data, including heart rate, blood pressure, weight, oxygen saturation, and temperature. Other members of the household or caregivers may monitor the health care process. The patients are triaged according to their vitals. The central station clinician is responsible for the initial interpretation of the data and contacts the patient with any health care concerns, including blood pressure changes, weight gain, or oxygen level fluctuations [3,4] (Table 1).

**Table 1:** Telemedicine services for chronic diseases and telerehabilitation at the Visiting Nurse Association.

Type of disease	Types of patient telehealth technology and interventions
Stroke rehabilitation and chronic heart disease	Devices for monitoring blood pressure, heart rate, oxygen saturation, and weight Tablet and wireless gateway for data transmission Web-based portal transmitting data from the patient blood pressure monitor and other devices to health care professionals Health care personnel include regional center operator, family practitioner, cardiologist, neurologist, and other health care professionals if needed
Chronic obstructive pulmonary disease	Pulse oximeter, weighing machine Tablet and wireless gateway for data transmission Web-based portal transmitting data from the pulse oximeter to health care professionals Data capturing readings and patient-selected questions Follow-up in person or by telephone Health care personnel include respiratory nurse, family practitioner, pulmonologist, and other health care professionals if needed

### Conclusion and Future Directions

Some patients recovering from stroke are stable; however, others have urgent needs. It is largely older adults with chronic diseases, especially stroke and heart disease, that benefit from telehealth interventions [3,8]. Nationwide telehealth service quality studies related to the care of patients recovering from stroke would have a more significant impact on research and the perceptions of rehabilitation and telehealth quality service [9,10]. Patients that are handicapped and impaired and unable to reside in their home environment are not included in telerehabilitation services provided by home care agencies [3,9]. It would be informative to study the benefits of telerehabilitation and the care of patients recovering from stroke within nursing homes. Feedback from health care professionals and physician specialists will help to refine the collaborative care efforts for this vulnerable patient population. Future studies of telehealth interventions and stroke management in home care will be important, given the need for social distancing during the current global health pandemic [11,12]. Due to the rapid spread of coronavirus disease (COVID-19), the provision of rehabilitation and stroke care may place health care workers in a position of vulnerability as they may acquire the virus and spread it [13,14]. Telehealth management of patients would reduce their risk of exposure to any infectious agent, whether during a pandemic or community outbreak [13,14]. Therefore, future strategies should consider expanding telerehabilitation services for patients while addressing barriers and solutions with medical staff, caregivers, and patients [14,15]. This may shed light on whether telerehabilitation can have a supportive role alongside standard rehabilitation care in patients poststroke and uncover the barriers and facilitators of this method of health care delivery.

## Abbreviations

COVID-19: coronavirus disease
VNA: Visiting Nurse Association
